# DNA Methylation at Core N-Methyl-D-Aspartate (NMDA) Receptor Genes Reveals a Glutamatergic Signature of Aging in Post-COVID Whole Blood With Implications for Long-COVID Neuropsychiatric Sequelae

**DOI:** 10.7759/cureus.112902

**Published:** 2026-07-18

**Authors:** Ngo Cheung

**Affiliations:** 1 Psychiatry, Cheung Ngo Medical Limited, Hong Kong, HKG

**Keywords:** brain fog, calcium signaling, cheung glutamatergic regimen, cognitive impairment, dna methylation, epigenetic aging, glutamate, grin1, long-covid, nmda receptor

## Abstract

Background

Cognitive symptoms after SARS-CoV-2 infection, often described as “brain fog,” remain difficult to measure objectively and are biologically heterogeneous. DNA methylation may provide a stable, blood-accessible layer of information linking post-COVID immune remodeling, biological aging, and neuropsychiatric vulnerability. We re-analyzed GSE247869, a whole-blood Illumina MethylationEPIC dataset from individuals sampled six months after COVID-19 infection, to identify age-associated methylation signals with translational relevance. The present analysis was designed to characterize age-associated methylation within this post-COVID cohort, not to establish a COVID-19-specific signature or biological age acceleration.

Methodology

This was a cross-sectional analysis of a single post-COVID cohort, with 94 samples included in the age models and no COVID-19-negative comparator included in the analyzed model. Processed beta values were aligned to metadata, converted to M-values, and modeled at each cytosine-phosphate-guanine (CpG) using ordinary least squares with age and sex as predictors. Differentially methylated positions were corrected by Benjamini-Hochberg false discovery rate (FDR). CpGs were mapped to genes using robust annotation and Illumina manifest fallback. Gene-level signals were integrated using a multi-evidence prioritization score that incorporated statistical strength, effect size, multi-CpG support, direction consistency, known epigenetic-clock membership, and curated pathway membership.

Results

Within this cohort, the analysis identified 3,467 age-associated CpGs at FDR < 0.05, with an overall hypomethylation bias but focal hypermethylation at canonical aging loci. In total, 11 of 12 reference clock CpGs were recovered, including *ELOVL2*,* FHL2*,* TRIM59*,* EDARADD*,* ASPA*, and* PDE4C*. The strongest exploratory signal was enrichment of glutamatergic/N-methyl-D-aspartate (NMDA) genes, including *GRIN1*,* GRIN2C*,* GRIN2D*,* GRM1*,* GRM5*, and* SLC17A7. GRIN1 *and* GRIN2C* had high integrated evidence scores and showed age-associated hypermethylation. The prioritized genes mapped interpretively to glutamatergic synapse, calcium signaling, and cAMP signaling pathways, although these complete KEGG pathways were not tested as formal enrichment categories.

Conclusions

This re-analysis recovered established age-associated CpGs and identified age-associated methylation enrichment near glutamatergic/NMDA genes within this post-COVID cohort. It cannot determine whether these signals are specific to COVID-19 infection, reflect accelerated biological aging, or relate to cognitive symptoms because no COVID-19-negative comparator or symptom-level cognitive phenotyping was included in the present analysis. The glutamatergic finding is hypothesis-generating, particularly because the curated set was small and no independent replication cohort was analyzed. Future longitudinal and case-control studies integrating *GRIN1*/*GRIN2C* methylation with cognitive and inflammatory phenotyping are needed. Glutamatergic and calcium-signaling pathways may be evaluated in appropriately designed mechanistic and intervention studies, including but not limited to hypotheses related to the Cheung Glutamatergic Regimen, only after independent validation and careful safety evaluation.

## Introduction

Post-COVID cognitive impairment: clinical burden and current challenges

Cognitive impairment is frequently reported following SARS-CoV-2 infection and is often described by patients as “brain fog,” although the term encompasses heterogeneous difficulties with attention, memory, processing speed, and mental stamina. Patients often describe slowed thinking, word-finding difficulty, poor attention, impaired working memory, and reduced mental stamina. In clinical settings, this cluster is commonly labeled “brain fog,” although the term covers several partially overlapping problems rather than one defined syndrome. Long-COVID reviews have emphasized that neurological and cognitive complaints can occur alongside fatigue, dysautonomia, sleep disturbance, pain, and exercise intolerance, making it difficult to identify one dominant mechanism in individual patients [1].

A major difficulty is the lack of objective biomarkers that can separate post-viral sequelae from pre-existing vulnerability, normal aging, psychiatric comorbidity, and medication effects. Standard clinical tests may miss subtle executive dysfunction, while brain imaging and routine blood tests often do not explain symptoms. The biology is also likely mixed [2]. Some patients may have persistent immune activation, others may have autonomic or vascular dysfunction, and others may have altered neurotransmission or impaired repair after acute illness. For this reason, a useful biomarker does not need to explain all long-COVID. It needs to identify a biologically coherent subgroup that can be tested and treated more rationally. The dataset used for the present analysis did not include symptom-level cognitive phenotyping, so any relationship between the methylation findings and individual cognitive symptoms remains untested.

Age is a further complication. Older adults are more likely to have cognitive vulnerability before infection, but younger adults can also develop disabling cognitive symptoms after COVID-19. The clinical question is therefore not simply whether patients are older, but whether post-COVID biology interacts with aging pathways in a way that exposes particular molecular systems. Epigenetic data are well suited to this question because DNA methylation captures both age-related and environmentally influenced signals.

Epigenetic aging in post-viral states

DNA methylation is one of the most studied epigenetic marks in aging. Earlier work showed that methylation at selected cytosine-phosphate-guanine sites (CpGs) can predict chronological age across tissues, and Horvath’s multi-tissue clock established that a weighted set of CpGs can estimate DNA methylation age across a broad range of tissues and cell types [3]. Later reviews and forensic studies confirmed that age-associated CpGs include both broadly reproducible markers and tissue-specific markers [4,5].

SARS-CoV-2 infection has added a new layer to this field. Several studies have reported altered DNA methylation or apparent biological age acceleration after COVID-19, although designs and tissues differ [6,7]. The primary dataset re-analyzed here, GSE247869, was generated from whole blood of 96 individuals sampled six months after COVID-19 infection. The original study compared these post-COVID samples with healthy controls and reported differential methylation, modest Horvath clock age acceleration, and increased epigenetic drift [8]. Although the source study included healthy controls, the present re-analysis used the post-COVID samples for within-cohort age modeling and did not include a COVID-19-negative comparator in the analyzed model.

It is important, however, to separate the two questions. A case-control epigenetic clock analysis can test whether a post-COVID group appears older than expected for chronological age, whereas a cross-sectional per-CpG model within post-COVID individuals tests which CpGs vary with chronological age within that cohort. The present analysis addresses the second question. It does not by itself establish COVID-19-driven acceleration or show that the identified signals differ from those expected during healthy aging. Its purpose is to characterize age-associated methylation structure within post-COVID blood and to identify candidate genes and pathways for independent follow-up.

Glutamatergic signaling, NMDA receptors, and cognitive vulnerability

Glutamate is the main excitatory neurotransmitter in the central nervous system. N-methyl-D-aspartate (NMDA) receptors are central to learning, memory, synaptic plasticity, and activity-dependent calcium signaling. These receptors are heterotetrameric ion channels that require the GluN1 subunit, encoded by *GRIN1*, together with GluN2 subunits encoded by *GRIN2A* through *GRIN2D*. Subunit composition changes receptor kinetics, localization, calcium permeability, and downstream signaling [9,10].

*GRIN1* is therefore a core structural and functional component of NMDA signaling. *GRIN2C* and *GRIN2D* are less ubiquitous than *GRIN2A* and GRIN2B, but they are biologically important because they confer distinct channel properties. Group I metabotropic glutamate receptors, especially *GRM1* and *GRM5*, do not form ion channels themselves but regulate intracellular calcium and modulate NMDA receptor function through G-protein-coupled pathways [11]. *SLC17A7* encodes vesicular glutamate transporter 1 (VGLUT1), a vesicular glutamate transporter required for packaging glutamate into synaptic vesicles [12].

Although these genes are usually discussed in the context of neurons, glutamate signaling is not confined to the brain. Immune cells can express glutamate receptors and respond to glutamate, and T-cell glutamate signaling has been reviewed as a direct neuroimmune interface [13]. This point matters for whole-blood methylation. A methylation signal near glutamatergic genes in blood should not be interpreted as direct evidence of brain methylation change. It may still be clinically informative if it reflects immune-cell regulatory states, shared neuroimmune programs, or systemic vulnerability linked to cognitive symptoms.

The Cheung Glutamatergic Regimen framework and unmet translational needs

The Cheung Glutamatergic Regimen (CGR) has been proposed in prior publications as one candidate multi-component approach aimed at modulating glutamatergic tone, NMDA-related signaling, synaptic plasticity, and related bioenergetic pathways using existing agents [14,15]. These prior publications provide a rationale for future hypothesis testing but do not establish efficacy for post-COVID cognitive impairment. In the present paper, CGR is considered only as one possible downstream hypothesis among several and is not evaluated or recommended.

The framework has been discussed in Alzheimer’s disease and psychiatric contexts [14,15]. Its prior disease-specific framing should not be taken to imply that the regimen is appropriate for the cohort analyzed here, and the present methylation analysis does not provide treatment-selection or treatment-response evidence.

For post-COVID cognitive impairment, the main translational barrier is not only treatment selection but patient selection. A potential research question is whether molecular markers could eventually identify biologically distinct subgroups, but no methylation marker can be considered clinically actionable based on this analysis alone. If a glutamatergic intervention is to be tested, a biomarker is needed to identify people whose biology plausibly involves glutamatergic dysregulation. The present re-analysis was designed with this problem in mind. The aim was to identify age-associated methylation changes in post-COVID blood that are reproducible enough to validate the analysis and coherent enough to generate a clinically testable hypothesis, with special attention to glutamatergic and NMDA receptor genes.

Despite progress in epigenetic clocks and long-COVID immunology, integrated prioritization of age-associated methylation changes that are both mechanistically meaningful and clinically actionable remains limited. Here, GSE247869 was re-analyzed using a multi-evidence scoring framework. The analysis first tested whether canonical epigenetic aging loci were recovered as a methodological validation step, then performed exploratory gene-level aggregation and pathway prioritization to identify candidate mechanisms. The central finding was an enrichment of glutamatergic/NMDA genes, which should be interpreted as an age-associated pattern observed within this post-COVID sample rather than as a post-COVID-specific signature.

This study had two objectives: (1) to confirm, as a methodological validation step, that established age-associated CpGs, including epigenetic-clock loci, could be recovered from GSE247869 using this pipeline; and (2) to perform exploratory, hypothesis-generating gene- and pathway-level prioritization of age-associated methylation signals in this post-COVID cohort. Because no COVID-19-negative comparator was included in the present analysis, the study cannot determine whether any signal is specific to COVID-19 infection or accelerated by it. It characterizes age-methylation associations within a post-COVID sample only. The potential relevance of the findings to CGR-type approaches is considered as one possible downstream hypothesis and was not tested as a treatment objective.

## Materials and methods

Dataset and preprocessing

**Figure 1:**
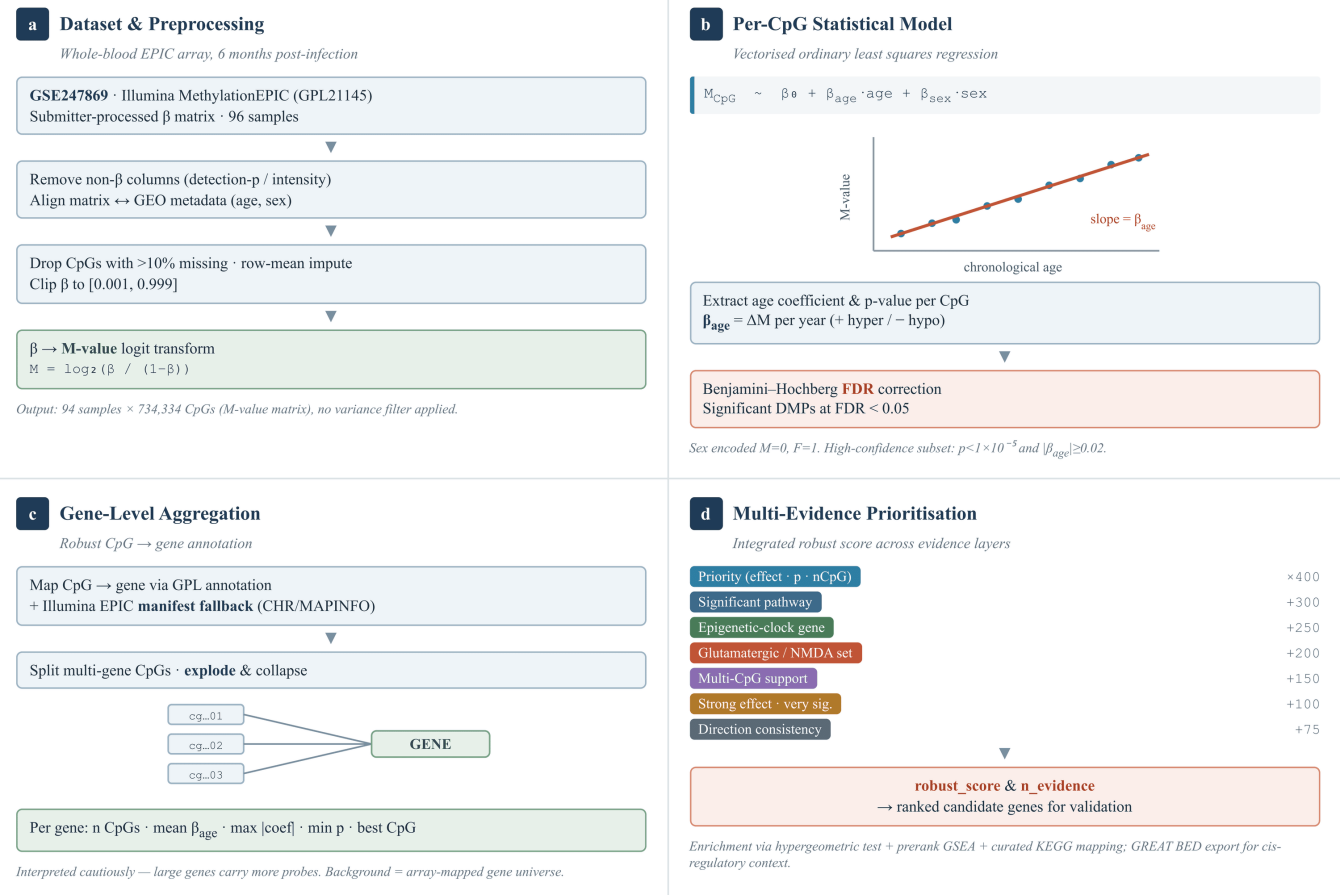
Analytical pipeline for age-associated DNA methylation in post-COVID whole blood (GSE247869). Four sequential stages of the re-analysis workflow. (a) Data acquisition and preprocessing; (b) per-CpG statistical modelling; (c) gene-level aggregation and annotation; (d) multi-evidence prioritization. The panels depict the methodology only. GSE247869 was originally generated and deposited by Calzari et al. [8]. Credits to Ngo Cheung. The image was generated using PowerPoint, and no artificial intelligence was used.

The analysis used GSE247869, a public Illumina Infinium MethylationEPIC whole-blood dataset from individuals assessed six months after COVID-19 infection [8]. This was a cross-sectional, single-cohort analysis; the present workflow did not include the COVID-19-negative controls used in the original study. The processed beta matrix was used as input (Figure 1). Non-beta columns, including detection p-value or intensity-like columns, were removed before modeling. Sample names in the methylation matrix were aligned to GEO sample metadata by GSM identifiers or sample titles where needed. The starting dataset contained 96 samples, of which 94 were modeled after metadata alignment and age filtering. The modeled age range was 35 to 92 years, with a mean age of 60.0 years and a standard deviation of 11.2 years. The sex distribution in the metadata was 71 male and 25 female samples.

CpGs with more than 10% missing values were removed. The remaining missing values were imputed by row mean. Row mean refers to the mean beta value for a given CpG across the available samples. Beta values were clipped to avoid boundary values and converted to M-values using a logit transformation. M-values were used for linear modeling because they are generally more suitable than beta values for regression-based methylation analyses. No variance filter was applied, so all retained CpGs were included. The number of input CpGs before the >10% missingness filter was not retained in the available analysis record; the final per-CpG analysis included 734,334 CpGs.

Statistical modeling

Each CpG was modeled using ordinary least squares regression with M-value as the dependent variable and chronological age and sex as predictors. The model was M-value as a function of intercept, age, and sex. Sex was encoded numerically as male equals 0, female equals 1, and unknown equals 0.5 if needed. The primary coefficient was the age coefficient, interpreted as a change in M-value per year of chronological age. A positive age coefficient indicated hypermethylation with increasing age, and a negative coefficient indicated hypomethylation with increasing age.

Multiple testing was controlled using Benjamini-Hochberg false discovery rate (FDR) correction. CpGs with age-term FDR below 0.05 were considered significant age-associated differentially methylated positions. A high-confidence subset was also defined using a nominal p-value below 1 × 10^−5^ and an absolute age coefficient at least 0.02 on the M-value scale.

Chip, plate, row, column, and position variables were not included in the reported model. The available analysis record did not document whether these technical variables were available in the source metadata or whether they had been formally tested.

Gene-level aggregation and annotation

CpGs were mapped to genes using available GPL annotation columns, with Illumina EPIC manifest fallback when gene names or genomic coordinates were missing. The exact release or version of the Illumina EPIC manifest used for fallback mapping was not specified in the available analysis record. Multi-gene annotations were split and expanded so that CpGs linked to more than one gene could contribute to each relevant gene-level entry. Gene-level summaries included the number of mapped CpGs, mean age coefficient, maximum absolute coefficient, minimum age p-value, and best CpG per gene.

This approach was chosen to reduce the risk of missing genes because of incomplete annotation fields. At the same time, gene-level results were interpreted cautiously because large genes naturally carry more array probes and can appear prominent simply because they have more measured CpGs.

Multi-evidence prioritization

Stage 3 prioritization integrated several independent evidence layers into a robust_score. The score included a normalized priority component based on CpG count, statistical strength, and effect size. Additional binary bonuses were assigned for membership in significantly enriched curated pathways, membership in the glutamatergic/NMDA set, known epigenetic-clock identity, multi-CpG support, strong effect size, high statistical significance, and direction consistency.

The weights were set as follows: normalized priority contributed up to 400 points; membership in a significant pathway added 300; glutamatergic/NMDA membership added 200; known epigenetic-clock identity added 250; multi-CpG support added 150; strong effect added 100; very significant p-value added 100; and direction consistency added 75. The n_evidence value counted the number of binary evidence flags present for each gene. This design intentionally favored genes supported by more than one kind of evidence rather than genes that were strong on a single metric only.

Because pathway and glutamatergic/NMDA membership were themselves components of robust_score, the score is not statistically independent of the enrichment analysis. The score was therefore used for prioritization and ranking rather than as independent confirmation of glutamatergic pathway enrichment.

Functional enrichment and pathway analysis

Curated gene-set enrichment was performed using hypergeometric testing against the array-specific mapped-gene universe, not the whole genome. This background was used to reduce bias from EPIC array probe coverage. The curated sets included known epigenetic-clock genes, glutamatergic/NMDA genes, synaptic signaling, mitochondrial oxidative phosphorylation, NAD/sirtuin aging, inflammation/immune genes, and senescence/cell-cycle genes.

The curated gene sets were author-defined. The complete original membership lists for the seven sets were not included in the available manuscript materials. This limits the exact reconstruction of the enrichment analysis until the full lists are deposited.

Gene-set enrichment analysis was also performed using gene-level ranked statistics. The exact per-gene ranking metric, permutation count, and random seed used for GSEA were not retained in the available analysis record. The GSEA results are therefore treated as exploratory and cannot be independently reproduced numerically from the present methods description alone.

KEGG pathway mapping was used to interpret prioritized genes in relation to glutamatergic synapse, neuroactive ligand-receptor interaction, calcium signaling, cAMP signaling, longevity regulation, PPAR signaling, fatty acid elongation, MAPK signaling, AMPK signaling, and synaptic vesicle cycle pathways. KEGG pathway identifiers were used for pathway naming and gene membership interpretation [16]. These KEGG mappings were interpretive; the complete KEGG pathways were not subjected to the same formal over-representation test used for the curated gene sets.

Directionality and multi-CpG analyses

For significant CpGs, genes were summarized by the number of hypermethylated and hypomethylated CpGs. Direction-consistent genes were defined by a net hyper-minus-hypo count of at least 3 or at most −3, with at least 5 significant CpGs. Multi-CpG candidate genes were also collected as potential regional methylation candidates. These were not treated as formal differentially methylated regions, because no adjacent-CpG DMR algorithm was applied.

For the exploratory GREAT export, 1,000 foreground regions and 734,334 background regions were generated. The exact rule used to select the 1,000 foreground regions was not retained, and no downloaded GREAT result file was available. Regulatory-domain enrichment was therefore not interpreted.

Statistical software and reproducibility

The pipeline was implemented in Python using pandas, NumPy, SciPy, statsmodels, matplotlib, seaborn, GEOparse, and gseapy. Exact Python and package version numbers were not retained in the available analysis record and therefore cannot be specified reliably here. Downstream and Stage 3 analyses used only pipeline output files and did not reload the large beta matrix. This allowed the integrated scoring and interpretation steps to be rerun efficiently and reproducibly from saved differential methylation, gene-level, enrichment, and directionality outputs.

The methylation dataset analyzed in this study is publicly available under GEO accession GSE247869 [8]. The complete analysis scripts, environment specification, full curated gene-set lists, and derived tables were not deposited in a public repository in the analysis record available for this manuscript. Additional analysis files may be made available upon reasonable request. The absence of a public, versioned code and gene-set repository is a limitation for exact independent reproduction of the downstream analyses.

Ethics approval and consent to participate

This study was based on secondary analysis of publicly available, de-identified DNA methylation data from GSE247869 [8]. No new human participants were recruited, no new biological samples were collected, and no identifiable personal data were used. Therefore, additional ethics approval and participant consent were not required for this re-analysis.

Competing interests

The author has developed and previously published on the CGR and is affiliated with a psychiatric clinic group in which related pharmacological approaches may be used. This relationship is disclosed for full transparency. CGR itself is not patented and is entirely free and open-sourced. The present work is an independent secondary analysis of publicly available data (GSE247869) and does not constitute a clinical recommendation or endorsement of any specific regimen.

## Results

Global age-associated methylation landscape

The per-CpG model identified 3,467 age-associated CpGs at FDR < 0.05 among 734,334 tested CpGs. Of these, 1,325 were hypermethylated with age, and 2,142 were hypomethylated with age. The hyper/hypo imbalance was strongly biased toward hypomethylation, with a binomial skew p-value of 4.57 × 10^−44^. This pattern is consistent with a common feature of aging methylomes: broad or regional loss of methylation with focal hypermethylation at selected regulatory loci [5,17]. These findings represent age associations within the analyzed cohort and should not be interpreted as COVID-19-specific differentially methylated positions. Table 1 summarizes the dataset, preprocessing, model, and global differential methylation results.

**Table 1:** Dataset, preprocessing, model, and global differential methylation results. Summary of the GSE247869 dataset, preprocessing workflow, statistical model, and global age-associated differential methylation results. GSE247869 was originally generated and deposited by Calzari et al. [8]. The absence of a COVID-19-negative comparator in the present analysis means that the table describes age-associated methylation within the analyzed post-COVID sample rather than a post-COVID-specific methylation signature. CpGs = cytosine-phosphate-guanine site; FDR = false discovery rate; DMP = differentially methylated position

Item	Value
Dataset	GSE247869
Study context	Whole-blood DNA methylation measured six months after COVID-19 infection; present analysis was cross-sectional and restricted to the post-COVID sample
Comparator in the present analysis	No COVID-19-negative comparator included
Platform	GPL21145, Illumina Infinium MethylationEPIC
Total samples	96
Samples modeled	94
Age range	35–92 years
Mean age	60.0 years
Age standard deviation	11.2 years
Sex distribution	71 male, 25 female
Tissue	Whole blood
Input matrix	Submitter-processed beta matrix
Non-beta columns removed	96 detection-p or intensity-like columns
Variance filtering	Off; all retained CpGs analyzed
Missing value handling	CpGs with more than 10% missing values removed; residual missing values imputed by row mean
Transformation	Beta values converted to M-values after clipping to 0.001–0.999
Input CpGs before missing value filtering	Not retained in the available analysis record
CpGs analyzed	734,334
CpGs mapped to genes	532,968 of 734,334
Unique cleaned gene universe	25,224 genes
Per-CpG model	M-value as a function of age and sex
Primary coefficient	Age coefficient, interpreted as change in M-value per year
Covariate	Sex encoded as male = 0, female = 1, unknown = 0.5
Technical covariates	Chip, plate, row, column, and position variables were not included; availability was not documented
Fallback manifest	Exact Illumina EPIC manifest release not retained in the available analysis record
Multiple-testing correction	Benjamini–Hochberg FDR
FDR-significant age DMPs	3,467 of 734,334 CpGs, 0.47%
Hypermethylated age DMPs	1,325
Hypomethylated age DMPs	2,142
Hyper/hypo skew	Hypomethylation-biased; binomial p = 4.57 × 10^−44^
Significant DMPs mapped to genes	2,474
High-confidence DMPs	100 CpGs; p < 1 × 10−5 and absolute coefficient at least 0.02
Top DMP shortlist	147 CpGs using relaxed threshold p < 1 × 10^−3^ and absolute coefficient above half threshold
Known-clock CpGs present	12
Known-clock CpGs significant at FDR < 0.05	11
*ELOVL2* CpGs with expected hypermethylation	3

The strongest individual age-associated CpGs included canonical epigenetic aging loci. The top CpG was cg16867657 in *ELOVL2*, with an age coefficient of 0.030590, p = 2.94 × 10^−31^, and FDR = 2.16 × 10^−25^. Additional top CpGs mapped to *ELOVL2*, *FHL2*, *KLF14*, *TRIM59*, *OTUD7A*, *NEFM*, and *GRIN1*. The recovery of *ELOVL2* was particularly important because methylation of this gene has been repeatedly identified as one of the most robust age markers in blood and other tissues [18,19]. The top age-associated CpGs are shown in Table 2.

**Table 2:** Top age-associated CpGs. Top CpG sites ranked by age-association strength using ordinary least squares regression of methylation M-value on chronological age and sex, followed by Benjamini–Hochberg false discovery rate correction. Positive coefficients indicate age-associated hypermethylation, and negative coefficients indicate age-associated hypomethylation. CpGs = cytosine-phosphate-guanine site; Chr = chromosome; Age coefficient = change in methylation M-value per year of chronological age; p-value = nominal age-term significance value; FDR = false discovery rate

CpG	Primary gene	Chr	Position	Age coefficient	P-value	FDR
cg16867657	ELOVL2	6	11044877	0.030590	2.938801e-31	2.158062e-25
cg24724428	ELOVL2	6	11044888	0.027796	1.009696e-19	3.707269e-14
cg04875128	OTUD7A	15	31775895	0.049958	3.529351e-18	8.639074e-13
cg17268658	FHL2	2	106015745	0.019445	5.021980e-17	8.403936e-12
cg21572722	ELOVL2	6	11044894	0.016300	5.722148e-17	8.403936e-12
cg06639320	FHL2	2	106015739	0.020890	2.093740e-16	2.562508e-11
cg22454769	FHL2	2	106015767	0.022744	2.702147e-16	2.834684e-11
cg03032497	Not mapped	14	61108227	0.020161	3.592337e-16	3.297469e-11
cg08097417	KLF14	7	130419133	0.022290	1.120892e-15	9.145657e-11
cg13206721	GPR158	10	25463350	0.020090	1.489984e-15	1.094146e-10
cg06210197	NEFM	8	24771256	0.026099	2.263467e-15	1.511037e-10
cg07553761	TRIM59	3	160167977	0.020679	3.294295e-14	2.015927e-09
cg11807280	Not mapped	2	66654644	-0.034711	5.087182e-14	2.873608e-09
cg15957394	AFAP1	4	7941823	0.017118	7.680243e-14	4.028474e-09
cg11667847	GRIN1	9	140033911	0.012366	2.935208e-13	1.418438e-08

Known clock CpG recovery provided a strong internal validation check. In total, 12 reference clock CpGs were present in the data, and 11 reached FDR < 0.05. These included CpGs mapped to *ELOVL2*,* FHL2*,* OTUD7A*,* TRIM59*,* EDARADD*,* CSNK1D*,* ASPA*,* and PDE4C*. Three *ELOVL2* CpGs showed the expected age-associated hypermethylation. This pattern supports that the age model was detecting genuine biological age-related variation rather than only technical noise. Known epigenetic-clock CpG recovery is shown in Table 3.

**Table 3:** Known epigenetic-clock CpG recovery. Recovery of reference epigenetic-clock CpGs using ordinary least squares regression of methylation M-value on chronological age and sex, followed by Benjamini–Hochberg false discovery rate correction. Recovery of clock CpGs was used as a methodological validation step and was not treated as an independent confirmation of the glutamatergic enrichment. CpGs = cytosine-phosphate-guanine site; Age coefficient = change in methylation M-value per year of chronological age; P-value = nominal age-term significance value; FDR = false discovery rate

CpG	Clock gene	Primary gene	Age coefficient	P-value	FDR
cg16867657	ELOVL2	ELOVL2	0.030590	2.938801e-31	2.158062e-25
cg24724428	ELOVL2	ELOVL2	0.027796	1.009696e-19	3.707269e-14
cg04875128	OTUD7A	OTUD7A	0.049958	3.529351e-18	8.639074e-13
cg21572722	ELOVL2	ELOVL2	0.016300	5.722148e-17	8.403936e-12
cg06639320	FHL2	FHL2	0.020890	2.093740e-16	2.562508e-11
cg22454769	FHL2	FHL2	0.022744	2.702147e-16	2.834684e-11
cg07553761	TRIM59	TRIM59	0.020679	3.294295e-14	2.015927e-09
cg09809672	EDARADD	EDARADD	-0.015578	2.043853e-07	6.413978e-04
cg19761273	CSNK1D	CSNK1D	-0.011019	5.842380e-07	1.263277e-03
cg02228185	ASPA	ASPA	-0.017508	3.359585e-06	3.783831e-03
cg17861230	PDE4C	PDE4C	0.016125	7.319834e-06	6.157163e-03
cg25809905	IGSF11	ITGA2B	-0.006314	3.849464e-02	4.898696e-01

High-confidence robust genes via integrated scoring

The Stage 3 prioritization shifted the analysis from individual CpGs to genes supported by multiple forms of evidence. The highest-ranked robust genes included *FHL2*,* EDARADD*,* ELOVL2*,* GRIN1*,* GRIN2C*,* PDE4C*,* IGSF11*,* OTUD7A*,* TRIM59*,* ASPA*,* GRIN2D*,* CSNK1D*,* SLC17A7*,* PENK*, and* KLF14*. Many of these are expected aging genes, but several are glutamatergic or synaptic genes that are less commonly emphasized in post-COVID methylation work. The leading Stage 3 prioritized genes are shown in Table 4.

**Table 4:** Leading Stage 3 prioritized genes for validation. Gene-level prioritization based on the Stage 3 multi-evidence scoring framework, incorporating ordinary least squares age-association results, effect size, multi-CpG support, direction consistency, epigenetic-clock membership, pathway membership, and glutamatergic/NMDA membership. CpGs = cytosine-phosphate-guanine site; Age coef. = mean age coefficient across mapped CpGs; Max abs. coef. = maximum absolute age coefficient; Minimum p = smallest nominal age-term p value; Robust score = integrated multi-evidence prioritization score; n evidence = number of binary evidence flags present; Dir. net = number of hypermethylated CpGs minus hypomethylated CpGs; P = significant pathway membership; C = known epigenetic-clock membership; G = glutamatergic/NMDA membership; M = multi-CpG support; S = strong effect size; V = very significant p-value; D = direction consistency

Rank	Gene	n CpGs	Age coef.	Max abs. coef.	Minimum p	Robust score	n evidence	Dir. net	Evidence flags
1	FHL2	203	0.001426	0.022744	5.021980e-17	989.974	6	3	P, C, M, S, V, D
2	EDARADD	70	-0.001722	0.016161	2.134616e-08	980.379	6	-4	P, C, M, S, V, D
3	ELOVL2	26	0.002875	0.030590	2.938801e-31	980.058	6	3	P, C, M, S, V, D
4	GRIN1	160	0.003667	0.021356	2.935208e-13	936.769	6	3	P, G, M, S, V, D
5	GRIN2C	39	0.002474	0.018863	3.055294e-07	928.253	6	3	P, G, M, S, V, D
6	PDE4C	140	0.001523	0.016125	7.319834e-06	909.648	5	2	P, C, M, S, V
7	IGSF11	96	-0.000373	0.015305	1.569889e-06	906.861	5	-1	P, C, M, S, V
8	OTUD7A	18	0.002307	0.049958	3.529351e-18	903.208	5	1	P, C, M, S, V
9	TRIM59	21	0.000956	0.020679	3.294295e-14	902.846	5	1	P, C, M, S, V
10	ASPA	26	-0.004489	0.017508	3.359585e-06	902.291	5	-2	P, C, M, S, V
11	GRIN2D	46	0.001168	0.019823	5.635035e-06	853.575	5	2	P, G, M, S, V
12	CSNK1D	116	0.000157	0.011019	5.842380e-07	808.187	4	-1	P, C, M, V
13	SLC17A7	36	0.002720	0.014732	5.701873e-06	752.903	4	1	P, G, M, V
14	PENK	50	0.010196	0.026983	8.359810e-10	729.279	5	5	P, M, S, V, D
15	KLF14	21	0.012413	0.037198	1.120892e-15	728.077	5	9	P, M, S, V, D

*FHL2* had the highest robust_score at 989.97 with n_evidence of 6. *EDARADD* had a robust_score of 980.38, also with n_evidence of 6. *ELOVL2* had a robust_score of 980.06 and n_evidence of 6. These genes mainly served as positive controls for age-related methylation biology. Their strong recovery showed that the model and annotation pipeline were functioning as expected.

The more exploratory signal came from *GRIN1* and *GRIN2C*. *GRIN1* had 160 mapped CpGs, an average age coefficient of 0.003667, a minimum p of 2.94 × 10^−13^, a robust_score of 936.77, and n_evidence of 6. *GRIN2C* had 39 mapped CpGs, an average age coefficient of 0.002474, a minimum p of 3.06 × 10^−7^, a robust_score of 928.25, and n_evidence of 6. *GRIN2D* also ranked highly, with a robust_score of 853.57 and n_evidence of 5. These scores summarize overlapping evidence categories and should not be interpreted as independent statistical confirmation of the glutamatergic pathway.

*PDE4C* ranked as another translationally relevant gene. It had 140 mapped CpGs, an average age coefficient of 0.001523, a minimum p of 7.32 × 10^−6^, a robust_score of 909.65, and n_evidence of 5. *PDE4C* is a known aging marker in forensic and blood methylation studies, but it is also biologically notable because PDE4 enzymes regulate cAMP signaling, a pathway involved in inflammation and synaptic function [20,21].

Glutamatergic/NMDA pathway as a standout signal

The strongest exploratory signal was enrichment of the curated glutamatergic/NMDA gene set. This set had six hits among the significant DMP genes: *GRIN1*,* GRIN2C*,* GRIN2D*,* GRM1*,* GRM5*, and* SLC17A7*. The fold enrichment was 5.57, with an FDR of 0.00135. The set contained only 15 genes in the array-specific universe, so the enrichment statistic is sensitive to the exact composition of this small curated list and should be interpreted as hypothesis-generating. This was one of only two curated sets reaching clear significance, the other being known epigenetic-clock genes. Curated gene-set enrichment and prioritized glutamatergic/NMDA genes are shown in Table 5 and Table 6.

**Table 5:** Curated gene-set enrichment using array-specific mapped-gene background. Curated gene-set enrichment was tested using a hypergeometric test against the EPIC array-specific mapped-gene universe, followed by Benjamini–Hochberg false discovery rate correction. The small size of the Glutamatergic_NMDA set makes its enrichment estimate sensitive to the gene-list composition. Query hits = number of significant differentially methylated position-associated genes overlapping the curated set; Fold enrichment = observed overlap divided by expected overlap; p-value = nominal hypergeometric enrichment p-value; FDR = false discovery rate; OXPHOS = oxidative phosphorylation; NAD = nicotinamide adenine dinucleotide; NMDA = N-methyl-D-aspartate

Gene set	Set size in the universe	Query hits	Fold enrichment	P-value	FDR	Overlap genes
Known_Epigenetic_Clock	11	11	13.920530	2.555794e-13	1.789056e-12	*ASPA*, *CSNK1D*, *EDARADD*, *ELOVL2*, *FHL2*, *IGSF11*, *KLF14*, *OTUD7A*, *PDE4C*, *PENK*, *TRIM59*
Glutamatergic_NMDA	15	6	5.568212	3.871821e-04	1.355137e-03	*GRIN1*, *GRIN2C*, *GRIN2D*, *GRM1*, *GRM5*, *SLC17A7*
Inflammation_Immune	13	3	3.212430	6.150326e-02	1.435076e-01	*NFKB1*, *SOCS3*, *TNF*
Synaptic_Signaling	12	2	2.320088	2.115358e-01	3.701877e-01	*BDNF*, *NRXN3*
Mitochondrial_OXPHOS	20	2	1.392053	4.263611e-01	5.969056e-01	*ATP5A1*, *OGDH*
Senescence_CellCycle	10	1	1.392053	5.255553e-01	6.131479e-01	GLB1
NAD_Sirtuin_Aging	16	1	0.870033	6.967276e-01	6.967276e-01	NADK

**Table 6:** Glutamatergic/NMDA genes prioritized in Stage 3. Stage 3 prioritized glutamatergic/*NMDA* genes based on ordinary least squares age-association results and the integrated multi-evidence scoring framework. NMDA = N-methyl-D-aspartate; CpGs = cytosine-phosphate-guanine site; Age coefficient = mean change in methylation M-value per year of chronological age across mapped CpGs; Minimum p-value = smallest nominal age-term p-value; Robust score = integrated multi-evidence prioritization score; n evidence = number of binary evidence flags present; Direction net = number of hypermethylated CpGs minus hypomethylated CpGs

Gene	n CpGs	Age coefficient	Minimum p-value	Robust score	n evidence	Direction net
GRIN1	160	0.003667	2.935208e-13	936.769	6	3
GRIN2C	39	0.002474	3.055294e-07	928.253	6	3
GRIN2D	46	0.001168	5.635035e-06	853.575	5	2
SLC17A7	36	0.002720	5.701873e-06	752.903	4	1
GRM1	137	0.001754	2.358846e-04	659.282	3	1
GRM5	60	0.000391	2.326181e-05	654.392	3	1

The gene-level detail showed a coherent direction. *GRIN1*,* GRIN2C*,* GRIN2D*,* GRM1*,* GRM5*, and* SLC17A7* all had positive average age coefficients in the pathway-specific summary. *GRIN1* had 43 significant or pathway-contributing CpGs in the enriched-set detail, with a mean coefficient of 0.002501 and a minimum p of 2.94 × 10^−13^. *GRIN2C* had 31 CpGs, with a mean coefficient of 0.003388 and a minimum p of 3.06 × 10^−7^. *GRIN2D* had 45 CpGs, with a mean coefficient of 0.001399 and a minimum p of 5.64 × 10^−6^. SLC17A7 had 34 CpGs, with a mean coefficient of 0.002637, and a minimum p of 5.70 × 10^−6^. *GRM5* and *GRM1* had *weaker* but still supportive signals.

The enrichment aligned directionally with the broader exploratory GSEA output, although the exact ranking statistic, permutation count, and random seed were not retained. Positively enriched terms included neuron differentiation (normalized enrichment score (NES) = 2.73), generation of neurons (NES = 2.65), neuropeptide signaling (NES = 2.30), and synapse assembly (NES = 2.26). The reported FDR q-values for these top terms were displayed as 0.0 in the source output and should be interpreted as rounded values rather than literal zero. Negative NES terms included olfactory transduction, sensory perception of chemical stimulus, asthma, *Staphylococcus aureus* infection, and autoimmune thyroid disease. The neuronal and synaptic terms were more consistent with the glutamatergic signal and were the main focus of interpretation. Additional GSEA, directionality, regional, and KEGG outputs are summarized in Table 7.

**Table 7:** Additional functional, directionality, regional, chromosomal, GREAT, and KEGG outputs. Summary of additional outputs from gene-set enrichment analysis, directionality testing, multi-CpG regional candidate screening, chromosomal enrichment testing, GREAT export, and KEGG interpretive mapping. Gene-set enrichment analysis used ranked gene-level statistics; chromosomal enrichment used over-representation testing followed by Benjamini–Hochberg false discovery rate correction. The KEGG entries in this table represent interpretive mapping of prioritized genes rather than formal enrichment tests of the complete pathways. GSEA = gene ontology, or gene-set enrichment analysis; GO = Gene Ontology; KEGG = Kyoto Encyclopedia of Genes and Genomes; GREAT = Genomic Regions Enrichment of Annotations Tool; BED = Browser Extensible Data format; NES = normalized enrichment score; FDR q value = false discovery rate-adjusted q value; CpGs = cytosine-phosphate-guanine site; chr = chromosome; observed/expected = observed-to-expected enrichment ratio

Output category	Finding	Key values
GSEA positive	GO neuron differentiation	NES = 2.729661; FDR q value = 0.000000
GSEA positive	GO generation of neurons	NES = 2.654279; FDR q value = 0.000000
GSEA positive	GO embryonic organ morphogenesis	NES = 2.401484; FDR q value = 0.000000
GSEA positive	GO neuropeptide signaling pathway	NES = 2.304514; FDR q value = 0.000000
GSEA positive	GO synapse assembly	NES = 2.260361; FDR q value = 0.000000
GSEA negative	KEGG olfactory transduction	NES = −3.093525; FDR q value = 0.000000
GSEA negative	GO sensory perception of chemical stimulus	NES = −2.693494; FDR q value = 0.000000
GSEA negative	GO sensory perception of smell	NES = −2.653727; FDR q value = 0.000000
GSEA negative	KEGG asthma	NES = −2.312169; FDR q value = 0.000000
Direction-consistent hypermethylation	KLF14	9 hypermethylated CpGs; 0 hypomethylated CpGs; net = 9
Direction-consistent hypermethylation	NEFM	8 hypermethylated CpGs; 0 hypomethylated CpGs; net = 8
Direction-consistent hypermethylation	ZIC1	7 hypermethylated CpGs; 0 hypomethylated CpGs; net = 7
Direction-consistent hypermethylation	PENK	5 hypermethylated CpGs; 0 hypomethylated CpGs; net = 5
Direction-consistent hypomethylation	NWD1	0 hypermethylated CpGs; 8 hypomethylated CpGs; net = −8
Direction-consistent hypomethylation	TAP2	0 hypermethylated CpGs; 5 hypomethylated CpGs; net = −5
Direction-consistent hypomethylation	TNF	0 hypermethylated CpGs; 5 hypomethylated CpGs; net = −5
Top multi-CpG regional candidate	CACNA1C	6,157 CpGs; age coefficient = −0.001903; minimum p = 5.259453e−04
Top multi-CpG regional candidate	PTPRN2	4,122 CpGs; age coefficient = −0.000762; minimum p = 6.067767e−06
Top multi-CpG regional candidate	MAD1L1	2,291 CpGs; age coefficient = 0.000318; minimum p = 3.355501e−04
Top multi-CpG regional candidate	CACNA1G	1,658 CpGs; age coefficient = −0.000994; minimum p = 4.472473e−10
Chromosomal distribution	No chromosome enriched at FDR < 0.05 with observed/expected ratio above 1	chr20 observed/expected = 1.26; p = 0.012716; FDR = 0.111613
GREAT export	Foreground and background BED files exported; foreground-selection rule not retained and downloaded GREAT result file unavailable	Foreground = 1,000 regions; background = 734,334 regions
KEGG interpretive mapping	Glutamatergic synapse, hsa04724	*GRIN1*,* GRIN2C*,* GRIN2D*,* GRM1*,* GRM5*,* SLC17A7*
KEGG interpretive mapping	Neuroactive ligand-receptor interaction, hsa04080	*GRIN1*,* GRIN2C*,* GRIN2D*,* GRM1*,* GRM5*,* PENK*,* SST*,* AVPR1A*,* NTSR2*
KEGG interpretive mapping	Calcium signaling pathway, hsa04020	*GRIN1*,* GRIN2C*,* GRIN2D*,* CACNA1G*,* ITPKB*
KEGG interpretive mapping	cAMP signaling pathway, hsa04024	*PDE4C*,* PENK*
KEGG interpretive mapping	Longevity regulating pathway, hsa04211	*ELOVL2*,* FHL2*,* KLF14*,* PDE4C*
KEGG interpretive mapping	Fatty acid elongation, hsa00062	ELOVL2

The glutamatergic pathway result is biologically notable because it links the curated-set finding to a system relevant to cognition and neuroimmune signaling. However, the convergence on glutamatergic synapse, calcium signaling, and cAMP signaling was based on interpretive KEGG mapping of prioritized genes rather than formal statistical enrichment of the complete KEGG pathways. It does not show that NMDA receptor methylation changed in the brain. It does show that age-associated methylation in post-COVID blood is enriched near genes central to glutamatergic signaling, especially *GRIN1* and *GRIN2C*.

Calcium signaling convergence

The glutamatergic signal converged with calcium signaling in the interpretive pathway mapping. NMDA receptors are ligand-gated calcium-permeable ion channels, and calcium entry through these receptors is central to synaptic plasticity, learning, and excitotoxic injury. In the same analysis, large multi-CpG genes included *CACNA1C* and *CACNA1G*, which encode voltage-gated calcium channel subunits. *CACNA1C* was the top multi-CpG candidate by CpG count, with 6,157 mapped CpGs, while *CACNA1G* had 1,658 mapped CpGs.

Stage 3 correctly downgraded *CACNA1C* compared with its Stage 2 ranking because its support was driven mainly by size and multi-CpG count. *CACNA1C* had a robust_score of 550.00 and n_evidence of 1, much lower than *GRIN1* or *GRIN2C*. This is an important safeguard. *CACNA1C* remains biologically interesting, especially because it is implicated in psychiatric disease and calcium-dependent neuronal signaling. Still, in this dataset, it should be treated as a DMR candidate requiring formal regional analysis rather than as a top discovery candidate.

*PTPRN2* was another large multi-CpG gene, with 4,122 mapped CpGs, a robust_score of 618.17, and n_evidence of 3. It is involved in dense-core vesicle biology and neuroendocrine secretion, and it may be relevant to vesicle-related signaling. However, as with *CACNA1C*, its size means that probe-density bias must be considered before giving it strong biological priority.

Direction-consistent and multi-CpG regional candidates

Direction-consistent genes provided a second way to identify stable signals. *KLF14* was the most consistently hypermethylated gene among significant CpGs, with nine hypermethylated and zero hypomethylated CpGs. *NEFM* had eight hypermethylated and zero hypomethylated CpGs. Other strongly hypermethylated genes included *ZIC1*,* PRRT1*,* CTNND2*,* ZNF518B*,* PCDHGA4*,* FAM19A4*,* GPR176*,* KCNQ1DN*,* NEUROD1*,* ZYG11A*,* PENK*, and *FEZF1*. *PENK* and *KLF14* were especially notable because they also ranked well in the robust gene prioritization.

The strongest hypomethylated genes included *NWD1*,* TAP2*,* TNF*,* FRMD4B*,* NAV2*,* PTPN7*,* FBLN1*,* LIMD1*,and *C20ORF54*. The presence of immune-related genes such as *TNF* and *TAP2* among consistently hypomethylated loci fits the broader possibility that age-associated methylation in post-COVID blood may reflect immune remodeling, antigen-presentation changes, or inflammatory tone. These findings are hypothesis-generating and require cell-composition-aware validation.

Chromosome-level testing did not identify any chromosome significantly enriched for age-associated DMPs after FDR correction. This suggests that the signal was not dominated by a single chromosomal artifact.

Absence of strong mitochondrial/NAD enrichment

A useful negative result was the lack of strong enrichment for mitochondrial oxidative phosphorylation or NAD/sirtuin aging genes. The curated mitochondrial oxidative phosphorylation set had two hits, *ATP5A1* and *OGDH*, with an FDR of 0.597. The NAD/sirtuin aging set had one hit, *NADK*, with an FDR of 0.697. These results do not argue against mitochondrial dysfunction in long-COVID or against bioenergetic components of CGR-related hypotheses. They only show that, in this specific whole-blood methylation dataset and this age-association model, mitochondrial and NAD/sirtuin genes were not dominant methylation signals.

The positive glutamatergic result should be treated with comparable caution. It was based on six genes from a 15-gene curated set, involved a modest sample size, and lacked independent replication. Both the positive and null pathway findings, therefore, require validation using larger cohorts, prespecified gene sets, and complementary molecular assays. These negative enrichment results are included in Table 5.

## Discussion

Validation of the aging model and interpretation of cross-sectional findings

The first conclusion from this re-analysis is that the age model behaved as expected. *ELOVL2*,* FHL2*,* TRIM59*,* EDARADD*,* ASPA*, and* PDE4C* were recovered among known epigenetic aging loci, and most reference clock CpGs reached FDR significance. This is important because a discovery signal is only useful if the underlying age model can recover established biology. *ELOVL2* hypermethylation, in particular, is one of the most reliable methylation markers of age across several settings [18,19]. Prior work also showed that *ELOVL2* and *C1orf132* retained age-prediction value across selected disease groups, supporting the idea that some methylation markers are robust to clinical heterogeneity [22].

The global directionality also fit aging methylomics. There were more hypomethylated than hypermethylated significant CpGs overall, yet the most prominent aging markers were focal hypermethylation events. This pattern is often seen in aging: broad methylation loss occurs alongside hypermethylation at selected CpG islands, promoters, or regulatory regions [5,17].

The main limitation is equally clear. This analysis modeled age association within a single post-COVID cohort at one six-month timepoint. It did not compare each individual’s methylation age with an expected value from a control population. Therefore, it cannot be established that these CpGs are accelerated by COVID-19. In addition, because no COVID-19-negative comparator was included in the present analysis, it is unknown whether the observed age associations differ from those expected in healthy aging or other post-viral states. The fact that the source study included controls does not remove this limitation because those controls were not used in the present per-CpG model. A proper acceleration analysis would require one or more epigenetic clocks, regression of DNA methylation age on chronological age, and comparison of residuals against controls or clinical subgroups. The present analysis should be read as a map of age-associated methylation structure within post-COVID blood, not as proof of accelerated aging or a COVID-19-specific methylation signature.

That said, cross-sectional age associations can still be informative. If a pathway is associated with age in a post-COVID sample, it may represent a candidate vulnerability or a feature of normal aging that deserves comparison with independent control cohorts. The most notable example here was the glutamatergic/NMDA pathway.

Mechanistic insights into glutamatergic/NMDA hypermethylation

**Figure 2:**
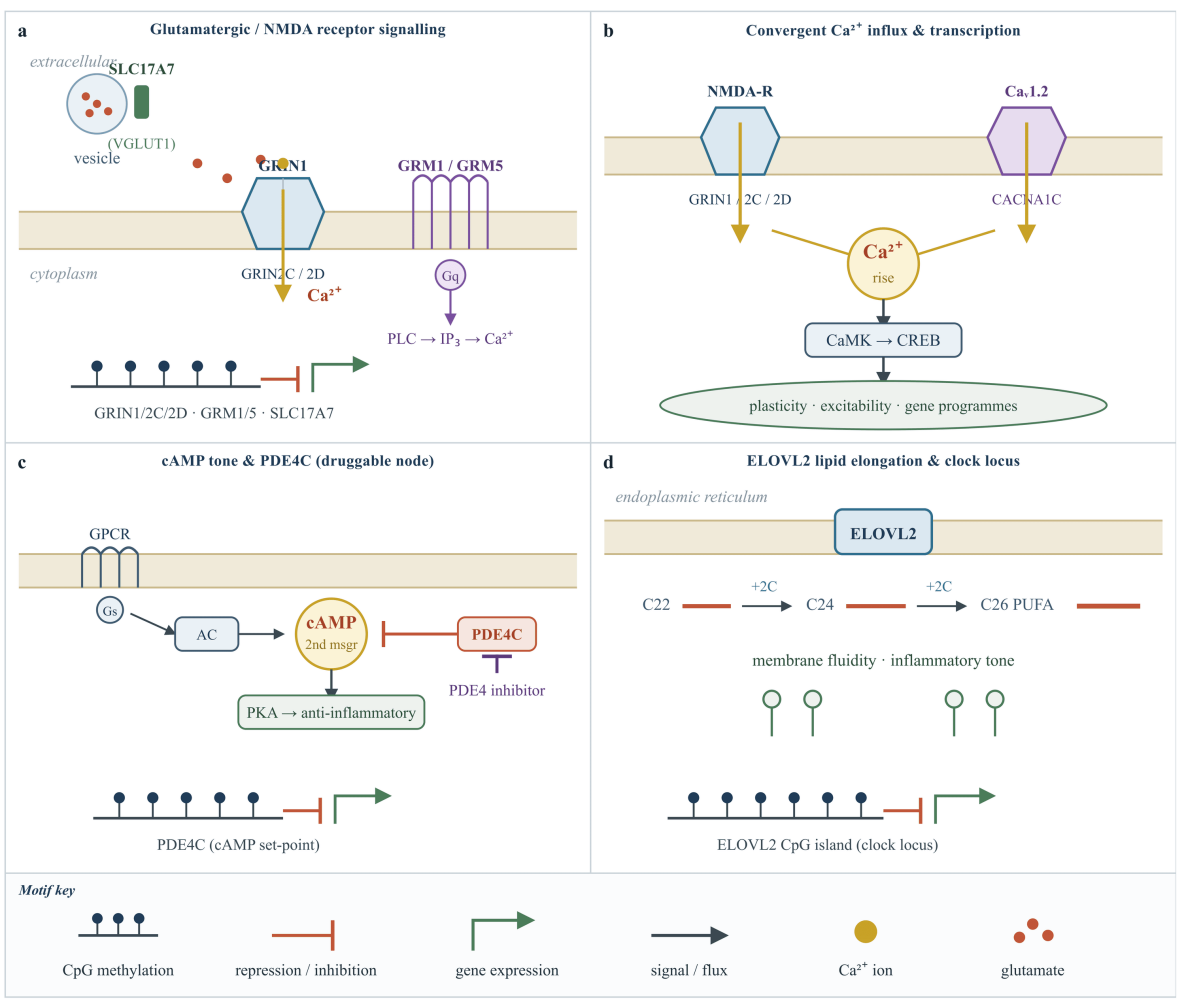
Proposed mechanistic axes linking age-associated DNA methylation to candidate biology. Schematics depict hypothesized molecular mechanisms only; no experimental data are shown. (a) Glutamatergic transmission. The vesicular transporter VGLUT1, encoded by *SLC17A7*, loads glutamate into synaptic vesicles; ionotropic NMDA receptors, containing obligatory *GRIN1* with *GRIN2C* or *GRIN2D*, act as ligand-gated calcium channels, while Gq-coupled metabotropic glutamate receptors *GRM1* and *GRM5* drive PLC–IP₃-dependent calcium mobilization. Filled lollipop symbols on gene tracks denote CpG methylation, and blunt-ended repression symbols denote predicted transcriptional repression. (b) Convergent calcium signalling. Calcium entering through NMDA receptors and the L-type calcium channel Caᵥ1.2, encoded by *CACNA1C*, converges on a shared cytosolic calcium rise that engages CaMK–CREB signalling to shape excitability, plasticity, and downstream gene programmes. (c) Cyclic-nucleotide tone. Gs-coupled receptors activate adenylyl cyclase to generate cAMP, promoting PKA-dependent anti-inflammatory signalling; PDE4C hydrolyzes cAMP and thereby sets its steady-state level. (d) Lipid-elongation clock locus. *ELOVL2* catalyzes iterative two-carbon elongation of very-long-chain polyunsaturated fatty acids, influencing membrane composition and inflammatory tone; CpG-island methylation of *ELOVL2* is an archetypal epigenetic-clock signal predicted to repress expression. All relationships are hypothesis-generating and require expression- and tissue-level validation. Credits to Ngo Cheung. The image was generated using PowerPoint, and no artificial intelligence was used.

NMDA receptor biology provides a plausible framework for interpreting the *GRIN* findings (Figure 2). Functional NMDA receptors require *GRIN1*, which encodes the GluN1 subunit. GluN2 subunits, encoded by *GRIN2A* through *GRIN2D*, shape receptor properties. *GRIN2C*- and *GRIN2D*-containing receptors often have different gating kinetics and signaling properties from *GRIN2A*- or *GRIN2B*-containing receptors [9,10]. These distinctions matter because NMDA receptor signaling can support synaptic plasticity under physiological conditions but contribute to excitotoxicity or maladaptive signaling when poorly regulated.

In this dataset, *GRIN1* and *GRIN2C* were among the highest-ranked non-clock genes. Both had an n_evidence of 6, and both were part of the significant glutamatergic/NMDA gene-set enrichment. *GRIN2D*,* GRM1*,* GRM5*, and* SLC17A7* added support. The pattern was not a single isolated CpG. It was a pathway-level signal involving ionotropic NMDA receptor subunits, metabotropic glutamate receptors, and vesicular glutamate transport. However, the multi-evidence score partly incorporated glutamatergic membership itself, so the score should not be regarded as an independent line of evidence beyond the underlying CpG-level and gene-set results.

One interpretation is that blood methylation near these genes reflects systemic neuroimmune signaling rather than brain tissue methylation. Immune cells can express glutamate receptors and respond to glutamate, and glutamate can influence T-cell behavior [13]. Post-COVID immune studies have reported persistent immune abnormalities months after infection, including activation of innate and adaptive immune compartments, altered interferon-associated signals, and T-cell dysregulation in long-COVID cohorts [23,24].

These observations provide several plausible but untested interpretations rather than a resolved biological mechanism. Whole-blood methylation at glutamatergic loci may reflect immune-cell regulatory programs that overlap with neuronal pathways or are influenced by chronic inflammatory signaling. Another possibility is cell-composition change: if certain immune cell types with distinct methylation profiles expand or contract with age after infection, pathway-level signals could emerge without methylation changing inside each cell type. Systemic inflammation, medication exposure, and other unmeasured post-COVID factors are additional possible explanations. Both the immune-cell and cell-composition explanations are compatible with the present data, and both are testable.

The potential clinical relevance is that glutamatergic signaling is related to cognition, but this analysis did not measure cognition, brain fog severity, or treatment response. NMDA receptors support learning and memory through synaptic plasticity, while abnormal glutamate signaling can impair network function. Long-COVID cognitive impairment may involve multiple mechanisms, but a glutamatergic subgroup is plausible, especially in patients with brain fog, slowed processing, fatigue-related cognitive collapse, or neuropsychiatric symptoms. The present findings do not establish such a subgroup. They provide candidate blood methylation markers with which that possibility could be tested in independent, clinically phenotyped cohorts.

Calcium signaling as a convergent node

Calcium signaling sits at the intersection of the *GRIN* and *CACNA* findings. NMDA receptors permit calcium influx when activated under appropriate voltage and ligand conditions. Group I metabotropic glutamate receptors can regulate intracellular calcium release and modulate NMDA receptor function. Voltage-gated calcium channels, including *CACNA1C* and *CACNA1G* products, regulate excitability and activity-dependent transcription. This makes calcium signaling a natural pathway-level bridge between synaptic plasticity, neuroinflammation, and psychiatric vulnerability. In this study, however, calcium signaling was identified through interpretive mapping of prioritized genes rather than formal enrichment testing of the complete pathway.

*CACNA1C* should be interpreted with restraint. It was a top multi-CpG gene, but its Stage 3 evidence count was low because the signal was largely driven by CpG number. Large genes with many probes are more likely to appear in array-based gene summaries. Still, *CACNA1C* is a biologically meaningful candidate because it has strong links to bipolar disorder, schizophrenia, depression, and neurodevelopmental syndromes [25,26]. If a formal DMR analysis confirms coherent age-related methylation across defined *CACNA1C* regions, it would strengthen the case for calcium signaling involvement.

*CACNA1G*, another voltage-gated calcium channel gene, also appeared as a multi-CpG candidate. Together with *GRIN1*, *GRIN2C*, and *GRIN2D*, these findings suggest that age-associated methylation in post-COVID blood may converge on calcium-dependent signaling. This remains a biologically plausible interpretation rather than a demonstrated pathway-level effect. It is relevant to cognitive symptoms because calcium signaling affects synaptic strength, neuronal survival, immune activation, and gene expression. It also links the methylation findings to the KEGG calcium signaling pathway hsa04020, which emerged as a biologically appropriate interpretive framework for the prioritized genes.

Potential translational relevance and how this addresses current difficulties

The most immediate potential use of these findings is as a hypothesis for biomarker-stratified research, not as a basis for clinical classification or treatment selection. Long-COVID cognitive impairment is clinically heterogeneous. A blood-based methylation marker at *GRIN1* or *GRIN2C* would not diagnose long-COVID, and it would not replace symptom assessment. The present study provides no diagnostic cutoff, prognostic threshold, or treatment-response association. Instead, such markers could be tested against cognitive measures, fatigue scales, neuropsychiatric symptoms, inflammatory markers, and treatment response in independent cohorts.

Because the author has published the CGR framework [14,15], its relevance should be stated transparently. CGR is one possible downstream pharmacological hypothesis among several, not a conclusion of the present analysis. The current data do not test CGR efficacy, identify patients who would benefit from it, or establish the safety of any component. Any future CGR-related study should be independently designed, prospectively registered, include prespecified clinical outcomes and appropriate controls, and undergo formal safety evaluation. The association observed here alone does not justify a treatment recommendation.

*PDE4C* provides a second potential translational axis. *PDE4C* is both a known epigenetic age marker and part of cAMP signaling. PDE4 inhibitors are already used or studied in inflammatory diseases, and apremilast has documented regulatory effects on innate immune pathways [27,21]. If *PDE4C* methylation or expression correlates with cognitive symptoms, inflammatory profiles, or treatment response, it could become a subject for independent mechanistic study. Again, the present data are not enough to support treatment selection, but they identify a testable target.

The multi-evidence scoring framework is also methodologically useful. Long-COVID research often struggles with long gene lists that are statistically significant but hard to interpret. By combining clock recovery, pathway enrichment, effect size, p-value strength, multi-CpG support, and direction consistency, this analysis prioritized genes that are less likely to be isolated artifacts. Because some of these components were also used to define pathway membership and the robust_score, the framework should be viewed as a prioritization aid rather than as an independent validation procedure. It also prevented over-prioritization of very large genes such as *CACNA1C* by distinguishing probe-count support from broader evidence support.

The KEGG pathways most relevant to interpretation were glutamatergic synapse hsa04724, calcium signaling hsa04020, cAMP signaling hsa04024, neuroactive ligand-receptor interaction hsa04080, fatty acid elongation hsa00062, and PPAR signaling hsa03320. These pathways were used to organize the biological interpretation of prioritized genes; they were not all formally enriched as complete pathways in the present analysis. These pathways map naturally onto the main findings: *GRIN* and *GRM* genes for glutamatergic synapse, *GRIN* and *CACNA* genes for calcium signaling, *PDE4C* for cAMP signaling, *ELOVL2* for fatty acid elongation, and *KLF14* for metabolic regulation.

Limitations

Several limitations must be kept in view. First, the design is cross-sectional. The age coefficient represents association with chronological age across individuals, not within-person aging speed. Claims of COVID-19-driven acceleration require clock-residual or longitudinal analysis.

Second, no COVID-19-negative comparator was included in the present analysis. Consequently, the study cannot determine whether the identified age-associated methylation patterns are specific to COVID-19 infection, related to post-COVID biology, or similar to age-associated patterns in healthy or otherwise ill populations. This is distinct from the limitation that the analysis cannot establish biological age acceleration.

Third, the tissue is whole blood. Blood methylation can reflect immune biology, systemic inflammation, and cell proportions, but it cannot be assumed to mirror brain methylation. This is especially important for genes usually considered neuronal, such as *GRIN1*,* GRIN2C*,* GRIN2D*,* GRM1*,* GRM5*, and* SLC17A7*. The appropriate framing is neuroimmune and systemic, not direct brain inference. The presence of a plausible immune-cell glutamate pathway does not resolve the tissue mismatch, because immune-cell signaling, cell-composition shifts, and systemic inflammatory effects were not directly measured.

Fourth, cell composition was not explicitly corrected in the provided model. Age, COVID-19 history, and long-COVID status can all influence immune cell proportions. Future analyses should estimate blood cell fractions or use reference-free deconvolution.

Fifth, multi-CpG gene summaries can be biased by gene size and probe density. *CACNA1C* and *PTPRN2* are biologically interesting, but their large CpG counts require formal DMR confirmation before strong conclusions are drawn.

Sixth, the Glutamatergic_NMDA gene set contained only 15 genes, with six overlapping genes producing the principal enrichment result. Hypergeometric statistics based on such a small, author-curated set can change substantially with the addition or removal of individual genes. The complete original membership lists were also not included in the available analysis materials, limiting independent assessment of set-definition sensitivity.

Seventh, the robust_score is not fully independent of the pathway findings because pathway and glutamatergic/NMDA membership contributed directly to the score. High scores for *GRIN1* and *GRIN2C*, therefore, should not be interpreted as separate confirmation of the enrichment result.

Eighth, the sample size was modest relative to the approximately 734,000 CpGs tested. The analysis was likely powered primarily to detect larger effects, whereas smaller-effect glutamatergic signals may be uncertain despite nominal or FDR-adjusted significance. The sex distribution was also imbalanced, with 71 male and 25 female samples. Sex was included as a covariate, but no sex-stratified sensitivity analysis was reported, and the study had limited power to assess sex-specific effects.

Ninth, the findings have not been replicated in an independent cohort. The absence of replication is particularly important because the study uses a single post-COVID sample, a small curated glutamatergic gene set, and a custom prioritization framework.

Tenth, downstream reproducibility is incomplete. Exact software versions, the EPIC manifest release, the complete curated gene-set lists, the GSEA ranking statistic, the permutation count, the random seed, the foreground-selection rule for GREAT, and a public code repository were not retained or available in the analysis record. These gaps reduce the extent to which the enrichment and prioritization results can be independently reproduced from the present text alone.

Eleventh, GREAT region-based enrichment was prepared but not completed. The pipeline generated foreground and background BED files using all tested CpGs as the background, which is the correct approach for controlling EPIC probe-design bias. However, no downloaded GREAT result file was available for Stage 3. Regulatory-domain interpretation, therefore, remains incomplete.

Finally, the analysis did not include symptom-level cognitive phenotypes, hospitalization severity, vaccination status, reinfection history, medication exposure, inflammatory markers, transcriptomics, proteomics, or metabolomics. These data would be needed to connect methylation findings to clinical outcomes.

Future directions

The next step should be an epigenetic clock residual analysis using Horvath, Hannum, PhenoAge, GrimAge, and newer immune-aware clocks, where possible. DNA methylation age should be regressed on chronological age, and residuals should be tested against post-COVID status, severity, and cognitive symptoms. This analysis should include an appropriate COVID-19-negative comparator and, ideally, an independent replication cohort. This would directly address acceleration.

Second, formal DMR calling should be performed for *GRIN1*,* GRIN2C*,* GRIN2D*,* CACNA1C*,* CACNA1G*,* PDE4C*, and* PTPRN2*. CpG-level results are useful, but regional methylation coherence would provide stronger biological support.

Third, *GRIN1* and *GRIN2C* methylation should be tested against cognitive outcomes. The most useful measures would include attention, working memory, processing speed, delayed recall, fatigue-related cognitive decline, and patient-reported brain fog severity.

Fourth, methylation should be integrated with gene expression. If age-associated hypermethylation at *GRIN* or *GRM* loci is associated with altered expression in immune cells, the biomarker argument becomes stronger. Single-cell methylation or sorted-cell validation would be especially valuable.

Fifth, the final prioritized genes should be overlapped with externally defined, prespecified glutamatergic, NMDA, mitochondrial, NAD, and immune gene sets. This would help determine whether the glutamatergic result is robust to changes in gene-set definition and would reduce dependence on a single author-curated list. The present analysis found strong glutamatergic/NMDA support but weak mitochondrial/NAD methylation enrichment. This suggests that CGR bioenergetic hypotheses may need to be tested using transcriptomics, metabolomics, or functional assays rather than relying only on blood methylation.

Finally, longitudinal validation is needed. A useful study would sample patients shortly after infection, at six months, and at one year, with parallel symptom measures and immune profiling. Such a design could determine whether GRIN methylation is stable, progressive, reversible, or treatment-responsive. Independent replication, open code and gene-set deposition, and prespecified analysis plans should be incorporated before any biomarker-guided intervention study is undertaken.

## Conclusions

This re-analysis of GSE247869 identified two principal findings within a single post-COVID whole-blood cohort. First, the age-associated methylation model recovered canonical epigenetic aging loci, including *ELOVL2*,* FHL2*,* TRIM59*,* EDARADD*,* ASPA*, and* PDE4C*. This provided a methodological validation check showing that recognizable age-associated methylation biology was detectable in the analyzed data. Second, the analysis identified enrichment of glutamatergic/NMDA genes, especially *GRIN1*,* GRIN2C*,* GRIN2D*,* GRM1*,* GRM5*, and* SLC17A7*. *GRIN1* and *GRIN2C* were among the strongest non-clock candidates by the integrated prioritization framework. These findings should be interpreted carefully. They do not prove that COVID-19 accelerated methylation aging, that the observed signals are specific to COVID-19, or that methylation changed in the brain. They also do not establish a relationship with cognitive symptoms because symptom-level phenotypes were not analyzed. The glutamatergic/NMDA result is a hypothesis-generating association that requires validation in larger, case-control and longitudinal cohorts using independent gene sets, formal regional analyses, cognitive phenotyping, cell-composition assessment, and gene-expression measurements. The findings support future studies integrating *GRIN1*/*GRIN2C* methylation with cognitive and inflammatory phenotyping. They also identify glutamatergic and calcium-signaling pathways as biologically plausible targets for mechanistic follow-up, although the KEGG convergence was interpretive rather than a formal enrichment result for the complete pathways. Any biomarker-stratified intervention study informed by these findings would require independent replication, prespecified clinical outcomes, appropriate controls, and careful safety evaluation. The present analysis is not a clinical recommendation or endorsement of the CGR or any other specific treatment.

## Appendices

**Table 8:** Top cytosine-phosphate-guanine (CpG) results. Chr = chromosome; FDR = false discovery rate

CpG	Primary gene	Chr	Position	Age coefficient	P-value	FDR
cg16867657	ELOVL2	6	11044877	0.030590	2.938801e-31	2.158062e-25
cg24724428	ELOVL2	6	11044888	0.027796	1.009696e-19	3.707269e-14
cg04875128	OTUD7A	15	31775895	0.049958	3.529351e-18	8.639074e-13
cg17268658	FHL2	2	106015745	0.019445	5.021980e-17	8.403936e-12
cg21572722	ELOVL2	6	11044894	0.016300	5.722148e-17	8.403936e-12
cg06639320	FHL2	2	106015739	0.020890	2.093740e-16	2.562508e-11
cg22454769	FHL2	2	106015767	0.022744	2.702147e-16	2.834684e-11
cg03032497	Not mapped	14	61108227	0.020161	3.592337e-16	3.297469e-11
cg08097417	KLF14	7	130419133	0.022290	1.120892e-15	9.145657e-11
cg13206721	GPR158	10	25463350	0.020090	1.489984e-15	1.094146e-10
cg06210197	NEFM	8	24771256	0.026099	2.263467e-15	1.511037e-10
cg07553761	TRIM59	3	160167977	0.020679	3.294295e-14	2.015927e-09
cg11807280	Not mapped	2	66654644	-0.034711	5.087182e-14	2.873608e-09
cg15957394	AFAP1	4	7941823	0.017118	7.680243e-14	4.028474e-09
cg11667847	GRIN1	9	140033911	0.012366	2.935208e-13	1.418438e-08
cg14692377	SLC6A4	17	28562685	0.018275	3.172409e-13	1.418438e-08
cg21990700	LOC283314	12	7260776	-0.019813	3.283716e-13	1.418438e-08
cg04478095	Not mapped	6	27599145	0.016461	1.137306e-12	4.639790e-08
cg22353329	CBX4	17	77814357	0.026587	1.725151e-12	6.667562e-08
cg20359994	Not mapped	6	27599141	0.015617	2.249003e-12	8.257598e-08
cg07955995	KLF14	7	130419159	0.037198	2.591469e-12	9.061923e-08
cg07502389	NEFM	8	24771259	0.019244	2.763041e-12	9.222704e-08
cg07171111	Not mapped	4	10462903	0.022191	4.641521e-12	1.481925e-07
cg24214068	NEFM	8	24771265	0.018635	5.704594e-12	1.745449e-07
cg01486610	RNF170	8	42749175	-0.019956	1.008242e-11	2.961546e-07

**Table 9:** Study, processing, and model summary. Note. Gene annotation used robust mapping with Illumina EPIC manifest fallback where required. CpGs = cytosine-phosphate-guanine site

Item	Value
Dataset	GSE247869
Study context	Whole-blood DNA methylation measured six months after COVID-19 infection
Platform	GPL21145, Illumina Infinium MethylationEPIC
Total samples	96
Samples modeled	94
Age range	35–92 years
Mean age	60.0 years
Age standard deviation	11.2 years
Sex distribution	71 male, 25 female
Tissue	Whole blood
Input matrix	Submitter-processed beta matrix
Non-beta columns removed	96 detection-p or intensity-like columns
Variance filtering	Off; all retained CpGs analyzed
Missing-value handling	CpGs with more than 10% missing values removed; residual missing values imputed by row mean
Transformation	Beta values converted to M-values after clipping to 0.001–0.999
CpGs analyzed	734,334
CpGs mapped to genes	532,968 of 734,334
Unique cleaned gene universe	25,224 genes

**Table 10:** Top clock-marker results. Note: Eleven of twelve reference clock CpGs were significant at FDR < 0.05. CpGs = cytosine-phosphate-guanine site; Chr = chromosome; FDR = false discovery rate

CpG	Clock gene	Primary gene	Age coefficient	P-value	FDR
cg16867657	ELOVL2	ELOVL2	0.030590	2.938801e-31	2.158062e-25
cg24724428	ELOVL2	ELOVL2	0.027796	1.009696e-19	3.707269e-14
cg04875128	OTUD7A	OTUD7A	0.049958	3.529351e-18	8.639074e-13
cg21572722	ELOVL2	ELOVL2	0.016300	5.722148e-17	8.403936e-12
cg06639320	FHL2	FHL2	0.020890	2.093740e-16	2.562508e-11
cg22454769	FHL2	FHL2	0.022744	2.702147e-16	2.834684e-11
cg07553761	TRIM59	TRIM59	0.020679	3.294295e-14	2.015927e-09
cg09809672	EDARADD	EDARADD	-0.015578	2.043853e-07	6.413978e-04
cg19761273	CSNK1D	CSNK1D	-0.011019	5.842380e-07	1.263277e-03
cg02228185	ASPA	ASPA	-0.017508	3.359585e-06	3.783831e-03
cg17861230	PDE4C	PDE4C	0.016125	7.319834e-06	6.157163e-03
cg25809905	IGSF11	ITGA2B	-0.006314	3.849464e-02	4.898696e-01

**Table 11:** Gene-set, pathway, and functional enrichment results (1). FDR = false discovery rate

Gene set	Set size in universe	Query hits	Fold enrichment	P-value	FDR	Overlap genes
Known_Epigenetic_Clock	11	11	13.920530	2.555794e-13	1.789056e-12	*ASPA*,* CSNK1D*,* EDARADD*,* ELOVL2*,* FHL2*,* IGSF11*,* KLF14*,* OTUD7A*,* PDE4C*,* PENK*,* TRIM59*
Glutamatergic_NMDA	15	6	5.568212	3.871821e-04	1.355137e-03	*GRIN1*,* GRIN2C*,* GRIN2D*,* GRM1*,* GRM5*,* SLC17A7*
Inflammation_Immune	13	3	3.212430	6.150326e-02	1.435076e-01	*NFKB1*,* SOCS3*,* TNF*
Synaptic_Signaling	12	2	2.320088	2.115358e-01	3.701877e-01	*BDNF*,* NRXN3*
Mitochondrial_OXPHOS	20	2	1.392053	4.263611e-01	5.969056e-01	*ATP5A1*,* OGDH*
Senescence_CellCycle	10	1	1.392053	5.255553e-01	6.131479e-01	GLB1
NAD_Sirtuin_Aging	16	1	0.870033	6.967276e-01	6.967276e-01	NADK

**Table 12:** Gene-set, pathway, and functional enrichment results (2). Note: The curated glutamatergic/NMDA set was enriched with FDR = 0.001355 and fold enrichment = 5.568. CpGs = cytosine-phosphate-guanine site

Gene	n CpGs	Age coefficient	Minimum p-value	Robust score	n evidence	Direction net
GRIN1	160	0.003667	2.935208e-13	936.769	6	3
GRIN2C	39	0.002474	3.055294e-07	928.253	6	3
GRIN2D	46	0.001168	5.635035e-06	853.575	5	2
SLC17A7	36	0.002720	5.701873e-06	752.903	4	1
GRM1	137	0.001754	2.358846e-04	659.282	3	1
GRM5	60	0.000391	2.326181e-05	654.392	3	1

**Table 13:** Gene-set, pathway, and functional enrichment results (3).

Direction	Term	NES	FDR q value
Positive	GO neuron differentiation	2.729661	0.000000
Positive	GO generation of neurons	2.654279	0.000000
Positive	GO embryonic organ morphogenesis	2.401484	0.000000
Positive	GO neuropeptide signaling pathway	2.304514	0.000000
Positive	GO synapse assembly	2.260361	0.000000
Positive	GO embryonic skeletal system morphogenesis	2.257117	0.000000
Positive	MSigDB hallmark pancreas beta cells	2.245306	0.000000
Positive	GO positive regulation of transcription regulatory region DNA binding	2.234620	0.000000
Negative	KEGG olfactory transduction	-3.093525	0.000000
Negative	GO sensory perception of chemical stimulus	-2.693494	0.000000
Negative	GO sensory perception of smell	-2.653727	0.000000
Negative	KEGG asthma	-2.312169	0.000000
Negative	KEGG *Staphylococcus aureus* infection	-2.179160	0.000895
Negative	KEGG autoimmune thyroid disease	-2.050099	0.002013
Negative	GO purine nucleoside bisphosphate metabolic process	-2.032824	0.059268
Negative	GO ribonucleoside bisphosphate metabolic process	-2.032824	0.059268

**Table 14:** Stage 3 prioritization and validation list. Note: Evidence flags are defined at the top of the appendix. Unlisted flags were false for that gene.

Rank	Gene	n CpGs	Age coef.	Max abs. coef.	Minimum p	Robust score	n evidence	Dir. net	Evidence flags
1	FHL2	203	0.001426	0.022744	5.021980e-17	989.974	6	3	P, C, M, S, V, D
2	EDARADD	70	-0.001722	0.016161	2.134616e-08	980.379	6	-4	P, C, M, S, V, D
3	ELOVL2	26	0.002875	0.030590	2.938801e-31	980.058	6	3	P, C, M, S, V, D
4	GRIN1	160	0.003667	0.021356	2.935208e-13	936.769	6	3	P, G, M, S, V, D
5	GRIN2C	39	0.002474	0.018863	3.055294e-07	928.253	6	3	P, G, M, S, V, D
6	PDE4C	140	0.001523	0.016125	7.319834e-06	909.648	5	2	P, C, M, S, V
7	IGSF11	96	-0.000373	0.015305	1.569889e-06	906.861	5	-1	P, C, M, S, V
8	OTUD7A	18	0.002307	0.049958	3.529351e-18	903.208	5	1	P, C, M, S, V
9	TRIM59	21	0.000956	0.020679	3.294295e-14	902.846	5	1	P, C, M, S, V
10	ASPA	26	-0.004489	0.017508	3.359585e-06	902.291	5	-2	P, C, M, S, V
11	GRIN2D	46	0.001168	0.019823	5.635035e-06	853.575	5	2	P, G, M, S, V
12	CSNK1D	116	0.000157	0.011019	5.842380e-07	808.187	4	-1	P, C, M, V
13	SLC17A7	36	0.002720	0.014732	5.701873e-06	752.903	4	1	P, G, M, V
14	PENK	50	0.010196	0.026983	8.359810e-10	729.279	5	5	P, M, S, V, D
15	KLF14	21	0.012413	0.037198	1.120892e-15	728.077	5	9	P, M, S, V, D
16	GRM1	137	0.001754	0.014215	2.358846e-04	659.282	3	1	P, G, M
17	GRM5	60	0.000391	0.014087	2.326181e-05	654.392	3	1	P, G, M
18	PTPRN2	4122	-0.000762	0.022906	6.067767e-06	618.169	3	1	M, S, V
19	LIMCH1	520	-0.001480	0.030319	4.088021e-06	459.404	4	-4	M, S, V, D
20	CACNA1G	1658	-0.000994	0.015173	4.472473e-10	458.637	3	1	M, S, V
21	CHFR	400	0.001104	0.020534	3.746654e-06	451.576	4	3	M, S, V, D
22	PEX5L	380	-0.000012	0.029484	2.226251e-06	450.341	4	4	M, S, V, D
23	TBCD	370	0.000252	0.018772	4.066674e-06	449.617	4	3	M, S, V, D
24	CTNND2	358	-0.000902	0.039565	6.575690e-06	448.906	4	6	M, S, V, D
25	AFAP1	284	0.000265	0.019808	7.680243e-14	444.875	4	3	M, S, V, D
26	PRUNE2	248	-0.000503	0.015284	8.535929e-06	441.648	4	3	M, S, V, D
27	CHST11	206	-0.000250	0.017758	4.216458e-08	439.182	4	-4	M, S, V, D
28	TRPS1	189	-0.001408	0.017878	5.404442e-08	438.067	4	-3	M, S, V, D
29	TAP2	181	-0.000787	0.016968	3.367172e-08	437.566	4	-5	M, S, V, D
30	CABYR	168	0.009295	0.020781	2.552150e-06	436.535	4	4	M, S, V, D
31	TEAD1	137	0.000507	0.022633	3.438638e-07	434.625	4	3	M, S, V, D
32	NR2F2	134	0.007845	0.019144	9.429278e-06	434.260	4	4	M, S, V, D
33	SHROOM3	129	-0.001601	0.016065	1.017584e-06	434.027	4	-3	M, S, V, D
34	CRMP1	123	0.002902	0.027919	2.248670e-07	433.759	4	3	M, S, V, D
35	GATA4	116	0.004457	0.024465	2.594320e-06	433.175	4	3	M, S, V, D
36	FAM123C	104	0.006898	0.022982	1.200402e-09	432.750	4	4	M, S, V, D
37	ITPKB	106	0.002459	0.015115	1.394822e-07	432.623	4	3	M, S, V, D
38	CPM	104	0.001133	0.015110	1.819196e-08	432.589	4	3	M, S, V, D
39	HNRNPUL1	106	-0.000599	0.015841	6.772299e-06	432.444	4	-3	M, S, V, D
40	FAM38A	95	0.000256	0.069570	4.240084e-07	432.092	4	-3	M, S, V, D
41	CALN1	98	-0.000277	0.022167	4.344354e-07	432.081	4	-3	M, S, V, D
42	WNT2B	97	0.002681	0.025951	1.695866e-06	431.968	4	3	M, S, V, D
43	CPLX2	97	0.004281	0.020357	7.771938e-06	431.872	4	3	M, S, V, D
44	CCDC50	94	0.002688	0.020276	4.380228e-07	431.812	4	4	M, S, V, D
45	SYNM	94	0.002729	0.016828	4.014114e-06	431.693	4	4	M, S, V, D
46	ZNF274	93	0.002394	0.021595	2.130172e-06	431.679	4	3	M, S, V, D
47	PRR16	91	-0.000184	0.029195	2.384753e-06	431.577	4	-4	M, S, V, D
48	C1RL	80	-0.001653	0.019813	3.283716e-13	431.564	4	-4	M, S, V, D
49	VWA3B	81	0.002206	0.024503	2.399226e-06	430.907	4	4	M, S, V, D
50	FOXP1	1230	0.000597	0.015462	1.292738e-06	430.482	3	-1	M, S, V
51	FAM19A4	72	0.003670	0.019666	2.119458e-07	430.416	4	5	M, S, V, D
52	ERCC1	73	-0.002186	0.019361	9.598059e-07	430.408	4	-3	M, S, V, D
53	NEFM	52	0.011337	0.026099	2.263467e-15	430.008	4	8	M, S, V, D
54	ASB2	65	0.000348	0.027991	1.543685e-06	429.904	4	3	M, S, V, D
55	RGS10	64	0.003683	0.019676	1.972304e-06	429.792	4	4	M, S, V, D
56	FRMD4B	61	-0.001139	0.021678	3.766650e-07	429.683	4	-5	M, S, V, D
57	FEZF1	62	0.009379	0.025324	4.872950e-06	429.644	4	5	M, S, V, D
58	NWD1	58	-0.002565	0.022049	1.270967e-07	429.541	4	-8	M, S, V, D
59	PPM1E	58	0.001411	0.015382	1.315721e-06	429.403	4	3	M, S, V, D
60	NINL	53	-0.000365	0.015525	1.337470e-09	429.403	4	3	M, S, V, D
61	ZIC1	53	0.008630	0.021608	3.553110e-07	429.167	4	7	M, S, V, D
62	TDRD9	53	0.000764	0.024497	2.875194e-06	429.081	4	4	M, S, V, D
63	LIMD1	46	-0.001122	0.015707	2.648214e-10	429.025	4	-4	M, S, V, D
64	CHGA	41	0.006128	0.024129	1.101060e-10	428.778	4	3	M, S, V, D
65	TRIL	37	0.008694	0.021255	6.343274e-10	428.424	4	4	M, S, V, D
66	ZYG11A	34	0.003192	0.024457	3.491229e-11	428.379	4	5	M, S, V, D
67	CACNG2	42	0.002835	0.018835	6.447243e-06	428.304	4	3	M, S, V, D
68	C11ORF85	38	0.004403	0.018314	8.970626e-07	428.135	4	4	M, S, V, D
69	STXBP5L	33	0.006330	0.017284	8.865806e-08	427.915	4	3	M, S, V, D
70	AVPR1A	31	0.007662	0.021276	6.232821e-07	427.711	4	3	M, S, V, D
71	ANKRD34C	31	0.004435	0.021975	1.669963e-06	427.667	4	4	M, S, V, D
72	BOK	23	0.003456	0.028531	2.435227e-10	427.591	4	3	M, S, V, D
73	SST	22	0.005025	0.015826	6.990092e-11	427.530	4	3	M, S, V, D
74	BMP8A	27	0.003483	0.019249	1.037329e-07	427.527	4	3	M, S, V, D
75	LOC100192378	29	0.007178	0.016243	3.665013e-06	427.476	4	3	M, S, V, D
76	SCGN	22	0.003935	0.015048	7.651624e-10	427.414	4	3	M, S, V, D
77	PRDM14	26	0.007354	0.023295	9.530568e-07	427.375	4	3	M, S, V, D
78	NEUROD1	23	0.009549	0.021992	7.345872e-08	427.295	4	5	M, S, V, D
79	ZNF518B	22	0.006792	0.027342	4.581549e-07	427.167	4	6	M, S, V, D
80	ERRFI1	23	0.003086	0.022049	7.075367e-06	427.081	4	3	M, S, V, D
81	ANKRD34B	18	0.003371	0.017222	4.838041e-09	427.078	4	3	M, S, V, D
82	NRIP3	13	0.003271	0.031334	3.076751e-11	427.052	4	3	M, S, V, D
83	VGF	18	0.005265	0.017959	3.322818e-07	426.882	4	3	M, S, V, D
84	CALB1	12	0.005399	0.016036	1.319912e-10	426.852	4	3	M, S, V, D
85	NTSR2	19	0.004683	0.025656	8.590569e-06	426.827	4	3	M, S, V, D
86	NKX2-4	18	0.006938	0.020175	4.083610e-06	426.774	4	3	M, S, V, D
87	C20ORF54	16	-0.005082	0.015640	3.975491e-06	426.626	4	-4	M, S, V, D
88	BST2	15	-0.009875	0.017163	1.939389e-06	426.601	4	-4	M, S, V, D
89	C13ORF33	14	0.007419	0.027263	2.302552e-06	426.572	4	3	M, S, V, D
90	SLC25A21-AS1	11	-0.005631	0.017775	3.481745e-08	426.533	4	-3	M, S, V, D
91	FOXE3	13	0.007355	0.025906	1.417020e-06	426.524	4	4	M, S, V, D
92	LOC375196	7	0.014968	0.031892	1.807974e-09	426.473	4	3	M, S, V, D
93	C6ORF155	11	0.013836	0.039393	9.087680e-06	426.365	4	4	M, S, V, D
94	PRDM16	1228	-0.001993	0.021554	2.269359e-05	405.244	3	-4	M, S, D
95	TRAPPC9	755	-0.000601	0.015061	9.910871e-07	399.659	3	2	M, S, V
96	MAD1L1	2291	0.000318	0.017573	3.355501e-04	399.102	2	0	M, S
97	CDH4	681	-0.001063	0.017811	3.413779e-06	394.809	3	-2	M, S, V
98	CDH13	676	-0.001300	0.015872	2.765298e-06	394.486	3	-2	M, S, V
99	KCNQ1	668	-0.001233	0.018171	7.534951e-07	394.038	3	-1	M, S, V
100	FAM107B	556	-0.000618	0.016946	2.974910e-06	386.698	3	-1	M, S, V

**Table 15:** Direction outputs. CpGs = cytosine-phosphate-guanine site

Direction group	Gene	Hypermethylated CpGs	Hypomethylated CpGs	Net	Total
Strong hyper	KLF14	9	0	9	9
Strong hyper	NEFM	8	0	8	8
Strong hyper	ZIC1	7	0	7	7
Strong hyper	PRRT1	7	0	7	7
Strong hyper	CTNND2	6	0	6	6
Strong hyper	ZNF518B	6	0	6	6
Strong hyper	PCDHGA4	6	0	6	6
Strong hyper	FAM19A4	5	0	5	5
Strong hyper	GPR176	5	0	5	5
Strong hyper	KCNQ1DN	5	0	5	5
Strong hyper	NEUROD1	5	0	5	5
Strong hyper	ZYG11A	5	0	5	5
Strong hyper	PENK	5	0	5	5
Strong hyper	FEZF1	5	0	5	5
Strong hypo	NWD1	0	8	-8	8
Strong hypo	TAP2	0	5	-5	5
Strong hypo	TNF	0	5	-5	5
Strong hypo	FRMD4B	0	5	-5	5
Strong hypo	NAV2	0	5	-5	5
Strong hypo	PTPN7	0	5	-5	5
Strong hypo	FBLN1	0	5	-5	5
Strong hypo	LIMD1	1	5	-4	6
Strong hypo	C20ORF54	1	5	-4	6

**Table 16:** Regional outputs. Note: These are candidate regional signals only; a formal DMR algorithm was not applied. CpGs = cytosine-phosphate-guanine site

Gene	n CpGs	Age coefficient	Minimum p-value
CACNA1C	6157	-0.001903	5.259453e-04
PTPRN2	4122	-0.000762	6.067767e-06
MAD1L1	2291	0.000318	3.355501e-04
ST3GAL3	1810	-0.000270	7.364052e-04
DYSF	1686	-0.000971	2.605481e-02
PDE9A	1660	0.000221	2.259055e-05
CACNA1G	1658	-0.000994	4.472473e-10
DISC1	1451	-0.000777	7.055676e-03
PRKAR1B	1364	0.000341	8.062851e-04
ABLIM2	1362	-0.000059	3.745565e-03
PARD3	1348	-0.002552	3.959935e-04
CUX1	1328	0.000023	3.463055e-03
TCF4	1302	-0.000068	2.467067e-03
COL13A1	1300	0.000353	5.235515e-04
TCF7L2	1281	-0.000788	1.798157e-02
SEPT9	1244	0.000999	7.866738e-04
FOXP1	1230	0.000597	1.292738e-06
PRDM16	1228	-0.001993	2.269359e-05
NRG1	1191	0.000739	1.766325e-04
KCNMA1	1174	-0.001563	6.494352e-05

**Table 17:** Chromosomal outputs. Note: No chromosome was enriched for significant age DMPs at FDR < 0.05 with an observed/expected ratio above 1. CpGs = cytosine-phosphate-guanine site; Chr = chromosome; FDR = false discovery rate

Chromosome	CpGs tested	Significant CpGs	Expected significant CpGs	Observed/expected	P-value	FDR
chr1	71481	370	337.5	1.10	0.080693	0.295875
chr2	56535	260	266.9	0.97	0.712699	0.825230
chr3	43327	248	204.6	1.21	0.003214	0.070712
chr4	31800	147	150.1	0.98	0.837930	0.877832
chr5	39128	201	184.7	1.09	0.223698	0.546817
chr6	46033	215	217.3	0.99	0.918739	0.918739
chr7	39417	175	186.1	0.94	0.440289	0.691883
chr8	33238	140	156.9	0.89	0.186557	0.513030
chr9	22850	98	107.9	0.91	0.359333	0.658777
chr10	36198	144	170.9	0.84	0.038354	0.168758
chr11	42944	191	202.8	0.94	0.438542	0.691883
chr12	39176	176	185.0	0.95	0.531053	0.730198
chr13	18228	90	86.1	1.05	0.665302	0.813146
chr14	25925	128	122.4	1.05	0.586760	0.759336
chr15	24766	142	116.9	1.21	0.023007	0.126539
chr16	32570	124	153.8	0.81	0.015220	0.111613
chr17	38872	169	183.5	0.92	0.300119	0.600237
chr18	13311	54	62.8	0.86	0.282547	0.600237
chr19	33100	159	156.3	1.02	0.809855	0.877832
chr20	20648	123	97.5	1.26	0.012716	0.111613
chr21	8987	33	42.4	0.78	0.165288	0.513030
chr22	15800	80	74.6	1.07	0.522862	0.730198

**Table 18:** Suggested KEGG interpretive mapping.

Tier	KEGG pathway	KEGG ID	Key genes from prioritized results	Interpretive relevance
Tier 1	Glutamatergic synapse	hsa04724	*GRIN1*,* GRIN2C*,* GRIN2D*,* GRM1*,* GRM5*,* SLC17A7*	Directly matches the strongest discovery pathway signal.
Tier 1	Neuroactive ligand-receptor interaction	hsa04080	*GRIN1*,* GRIN2C*,* GRIN2D*,* GRM1*,* GRM5*,* PENK*,* SST*,* AVPR1A*,* NTSR2*	Captures neurotransmitter receptor and peptide signaling genes in the prioritized list.
Tier 1	Calcium signaling pathway	hsa04020	*GRIN1*,* GRIN2C*,* GRIN2D*,* CACNA1G*,* ITPKB*	Links NMDA receptor and calcium-channel biology.
Tier 1	cAMP signaling pathway	hsa04024	*PDE4C*,* PENK*	Highlights cyclic nucleotide signaling and druggable PDE biology.
Tier 2	Longevity-regulating pathway	hsa04211	*ELOVL2*,* FHL2*,* KLF14*,* PDE4C*	Provides aging-context interpretation for validated clock-like genes.
Tier 2	PPAR signaling pathway	hsa03320	KLF14	Connects metabolic and inflammatory regulation to age-associated methylation.
Tier 2	Fatty acid elongation	hsa00062	ELOVL2	Anchors the strongest canonical clock gene to lipid metabolism.
Tier 3	MAPK signaling pathway	hsa04010	*GRIN* genes, calcium-related genes	Peripheral but plausible downstream signaling context.
Tier 3	AMPK signaling pathway	hsa04152	General aging and metabolism candidates	Relevant to aging biology but not a dominant methylation signal in this dataset.
Tier 3	Synaptic vesicle cycle	hsa04721	*SLC17A7*,* CPLX2*,* STXBP5L*	Supports vesicle and synaptic-release interpretation.
